# Characterizing viral species in mosquitoes (Culicidae) in the Colombian Orinoco: insights from a preliminary metagenomic study

**DOI:** 10.1038/s41598-023-49232-9

**Published:** 2023-12-12

**Authors:** Marcela Gómez, David Martínez, Luisa Páez-Triana, Nicolás Luna, Jorge Luis De las salas, Carolina Hernández, Alexander Zamora Flórez, Marina Muñoz, Juan David Ramírez

**Affiliations:** 1 Centro de Investigaciones en Microbiología y Biotecnología-UR (CIMBIUR), Facultad de Ciencias Naturales, Universidad del Rosario, Bogotá, Colombia; 2 Grupo de Investigación en Ciencias Básicas (NÚCLEO), Facultad de Ciencias e Ingeniería, Universidad de Boyacá, Tunja, Colombia; 3 Secretaría Departamental de Salud del Vichada, Puerto Carreño, Colombia; 4 Molecular Microbiology Laboratory, Department of Pathology, Molecular and Cell-Based Medicine, Icahn School of Medicine at Mount Sinai, New York, NY USA

**Keywords:** Virology, Metagenomics

## Abstract

Mosquitoes (Diptera: Culicidae) are primary vectors of arthropod-borne viruses (arboviruses) that pose significant public health threats. Recent advances in sequencing technology emphasize the importance of understanding the arboviruses and insect-specific viruses (ISVs) hosted by mosquitoes, collectively called the “virome”. Colombia, a tropical country with favorable conditions for the development and adaptation of multiple species of Culicidae, offers a favorable scenario for the transmission of epidemiologically important arboviruses. However, entomovirological surveillance studies are scarce in rural areas of the country, where humans, mosquitoes, and animals (both domestic and wild) coexist, leading to a higher risk of transmission of zoonotic diseases to humans. Thus, our study aimed to perform a preliminary metagenomic analysis of the mosquitoes of special relevance to public health belonging to the genera *Ochlerotatus*, *Culex*, *Limatus*, *Mansonia*, *Psorophora*, and *Sabethes*, within a rural savanna ecosystem in the Colombian Orinoco. We employed third-generation sequencing technology (Oxford Nanopore Technologies; ONT) to describe the virome of mosquitoes samples. Our results revealed that the virome was primarily shaped by insect-specific viruses (ISVs), with the Iflaviridae family being the most prevalent across all mosquito samples. Furthermore, we identified a group of ISVs that were common in all mosquito species tested, displaying the highest relative abundance concerning other groups of viruses. Notably, *Hanko iflavirus-1* was especially prevalent in *Culex eknomios* (88.4%) and *Ochlerotatus serratus* (88.0%). Additionally, other ISVs, such as *Guadalupe mosquito virus* (GMV), *Hubei mosquito virus1* (HMV1), *Uxmal virus*, *Tanay virus*, *Cordoba virus*, and *Castlerea virus* (all belonging to the *Negevirus* genus), were found as common viral species among the mosquitoes, although in lower proportions. These initial findings contribute to our understanding of ISVs within mosquito vectors of the Culicidae family in the Eastern Plains of Colombia. We recommend that future research explore deeper into ISV species shared among diverse vector species, and their potential interactions with arboviruses. In addition, we also showed the need for a thorough exploration of the influence of local rural habitat conditions on the shape of the virome in mosquito vectors.

## Introduction

Mosquitoes (Diptera, Culicidae) are globally recognized vectors of medically and veterinary significant pathogens, contributing to millions of disease cases annually^1^. Hematophagous mosquito species play a crucial role in transmitting various epidemic arboviruses, particularly those belonging to the Flaviviridae and Togaviridae families^2–4^. In tropical and subtropical regions, mosquitoes capable of transmitting pathogenic viruses in both urban and rural cycles are primarily found within the *Aedes* and *Culex* genera^5–7^. These vectors serve as primary carriers of viruses such as dengue virus (DENV), Zika virus (ZIKV) West Nile virus (WNV)^8^, St. Louis encephalitis (SLEV) and Japanese encephalitis (JEV) from the Flaviviridae family, as well as chikungunya virus (CHIKV) from the Togaviridae family. The biological characteristics of *Aedes and Culex* mosquitoes facilitate their rapid adaptation to new habitats and their wide geographic expansion, often associated with anthropogenic interventions in forested areas,^9–11^ unplanned urbanization^12,13^, global trade, and climate change^14,15^. Additionally, other members of the Culicidae family, belonging to the genera *Haemagogus, Sabethes, Mansonia* and *Psorophora,* have been implicated in the transmission of viruses such as yellow fever virus (YFV), Mayaro virus (MAYV)^16,17^ and Venezuelan equine encephalitis virus (VEEV)^8,18^ in sylvatic cycles. However, in rural areas at risk of zoonotic disease transmission to humans (spillover), the vectors endemic to these areas remain poorly studied.

The importance of developing new entomovirological surveillance strategies to monitor (re)emerging viruses and discover novel ones has been propelled by advancements in next-generation sequencing (NGS) technologies^19,20^. These breakthroughs have driven the progress of viral metagenomic studies in mosquitoes, leading to the identification of numerous viruses. This development has expanded our understanding of the diversity, taxonomy, and wide range of environmental conditions in which viral agents exist in mosquito vectors^19,20^. In addition to detecting arboviruses, a representative group in the mosquito virome, known as insect-specific viruses (ISVs)^21^, has been found. ISVs are described as viruses that naturally infect arthropods but cannot replicate in vertebrate cells or infect humans^20,22,23^ suggesting their long-term symbiotic interactions with their hosts^24^. Indeed, it has been proposed that ISVs play an important role in modulating vectorial competence^21,22,25,26^, and could be a key component in the development of new strategies for arbovirus control and the development of vaccines or diagnostic platforms^23,25,27^.

Current explorations into the mosquito virome have shed light on the concept of a “core virome,” which comprises a group of viruses (ISVs) shared among mosquito vector populations of the same species^20,24,28^. However, viruses shared between different mosquito species have also been detected^29–31^, probably due to environmental factors such as food resources and breeding sites^5,32^. In both cases, ISVs could directly affect the ecology and evolution of the mosquito host, as well as the rest of the mosquito’s microbiota^24^. Analyses of the global composition and distribution of the mosquito virome suggest that the environment, modes of virus transmission, and genetic factors in specific mosquito species or genera are factors that shape the virome composition^5^. Similarly, other studies show that viral dynamics depends primarily on interactions between mosquitoes and their environment, where feeding is considered, a critical factor affecting virus composition^29,30^ However, the factors influencing the complex associations of virome composition remain largely unknown, especially in mosquitoes associated with sylvatic cycles, in environmentally conserved natural areas.

Colombia, a world-renowned megadiverse nation, has a rich variety of mosquito species^33^.This diversity, coupled with the diversity of ecosystems, creates favorable conditions in approximately 85% of the country for the migration, adaptation, and proliferation of mosquitoes, which contributes to the increased prevalence of mosquito-borne diseases in the country^34–36^. The high richness of mosquitoes in different regions of the country is probably associated with landscape modifications, deforestation and changes in temperature^8,34^ favoring the proximity of wildlife vectors to humans, which increases the risk of transmission of known and emerging zoonotic arboviruses^37,38^. In the country, entomovirological surveillance efforts have focused mainly on viral species of public health importance^36,38^, especially in urban centers, while exploration of other viral species remains limited. In addition, a considerable portion of the viral composition in vectors and other insects associated with the family Culicidae remains unknown, highlighting the need for comprehensive research and studies in this field^34^. Findings on the virome of mosquito vectors in the country are an emerging and recently developed topic. A pioneering study employed a meta-taxonomic approach in *Aedes aegypti* and *Aedes albopictus* mosquitoes captured in urban areas in the center of the country, revealing significant differences in the presence, diversity, and abundance of viruses between these two mosquito species^39^. Another study conducted in a rural area in the northern (coastal) part of the country discovered a putative new virus called *Guachaca virus* (family Tymoviridae) in *Culex* spp. Phylogenetically related to plants, suggesting an ecological relationship between plant viruses and plant-feeding insects, as well as a selection pressure driving the adaptation of the virus in the mosquito vector^40^.

The Colombian Orinoco region, commonly known as the Eastern Plains, holds significant strategic importance owing to its exceptional biodiversity and extensive water resources. Despite documented cases of mosquito-borne diseases such as dengue, chikungunya, Zika, and yellow fever infections, this area has been relatively underexplored from an entomovirological perspective^35,36^. Rural areas in this region have experienced minimal human interference, with limited anthropogenic disturbance. This circumstance raises the possibility that potentially pathogenic viruses generate spillover events under deforestation scenarios^41^. Based on the results of a recent study focused on understanding the ecology and transmission dynamics of flaviviruses in mosquitoes (Diptera: Culicidae) from the Eastern Plains of Colombia^41^, the circulation of West Nile Virus (WNV) in *Culex browni* was revealed, highlighting the potential impact of human activities in these anthropogenically sensitive ecosystems^41^. Therefore, the objective of this study was to characterize the virome of mosquito vector species such as *Ochlerotatus serratus, Culex eknomios, Cx. browni, Limatus durhamii, Mansonia indubitans, Psorophora albipes, Psorophora ferox* and *Sabethes chloropterus*, previously identified in the ecosystem of the Orinoco River basin. The results obtained underline the importance of insect-specific viruses (ISVs), given their abundance and the presence of shared viral species among different mosquito hosts. We propose that habitat-related factors could shape mosquito virome composition, especially among mosquitoes coexisting in a local natural ecosystem with similar ecological characteristics.

## Results

### Metagenomic analysis of mosquitoes virome

From the genetic material (RNA) of adult vector mosquitoes identified (based on the combination of morphology and DNA barcoding) in a savanna ecosystem in the Orinoco region (Colombia)^41^, we selected eight of the most abundant and epidemiologically significant mosquito species belonging to six genera: *Ochlerotatus, Culex, Limatus, Mansonia, Psorophora and Sabethes*. The samples were organized into 15 pools, subjected to viral enrichment using SMRT-9 methodology and sequenced using Oxford Nanopore technology (Table 1). Subsequently, the viral metagenomic data was analyzed using different bioinformatics tools, revealing a total of 1,439,457 reads with an average of approximately 95,964 reads per sample. After removing bacterial sequences using the Minimap2 tool against the Silva 16S bacterial reference database, a total of 1,161,300 clean reads were obtained, showing a length of 375 bp and an average quality score of 9.5. Among them, 1408 sequences (1.88% of the total reads) were identified as viral per sample (Table 1).

**Table 1:** Summary of quality data and analysis of metagenomic sequencing results detected in field-collected mosquitoes from a local ecosystem in the Orinoco region of Colombia.

Pool (Sample)	Mosquito Species	Number of raw reads	Classified reads		Unclassified reads		Viral reads		Mean read length	Mean read quality
			Number	%	Number	%	Number	%		
Pool1	*Ochlerotatus serratus*	68,106	1563	2.29	66,543	97.70	1563	2.29	366.1	9.7
Pool2	*Ochlerotatus serratus*	56,280	1343	2.39	54,937	97.60	1343	2.39	370.8	9.7
Pool3	*Ochlerotatus serratus*	54,250	1501	2.77	52,749	97.20	1501	2.77	364	9.8
Pool4	*Ochlerotatus serratus*	77,745	2173	2.80	75,572	97.20	2173	2.80	368	9.8
Pool5	*Ochlerotatus serratus*	79,786	1825	2.29	77,961	97.70	1825	2.29	368.6	9.7
Pool6	*Ochlerotatus serratus*	74,223	1626	2.19	72,597	97.80	1626	2.19	382.6	9.7
Pool7	*Ochlerotatus serratus*	75,060	2115	2.82	72,945	97.20	2115	2.82	373	9.8
Pool8	*Culex eknomios*	59,225	815	1.38	58,410	98.60	815	1.38	400.3	9.7
Pool9	*Culex browni*	76,593	921	1.20	75,672	98.80	921	1.20	372.5	9.7
Pool10	*Limatus durhamii*	84,940	1007	1.19	83,933	98.80	1007	1.19	385.6	9.7
Pool11	*Mansonia indubitans*	119,658	1501	1.25	118,157	98.70	1501	1.25	386.7	9.7
Pool12	*Psorophora albipes*	64,698	951	1.47	63,747	98.50	951	1.47	380.5	9.8
Pool13	*Psorophora ferox*	89,835	1165	1.30	88,670	98.70	1165	1.30	382.9	9.8
Pool14	*Psorophora ferox*	90,583	1344	1.48	89,239	98.50	1344	1.48	374.4	9.8
Pool15	*Sabethes chloropterus*	90,318	1265	1.40	89,053	98.60	1265	1.40	345	9.6

### Characterization of viral families

**Figure 1:**
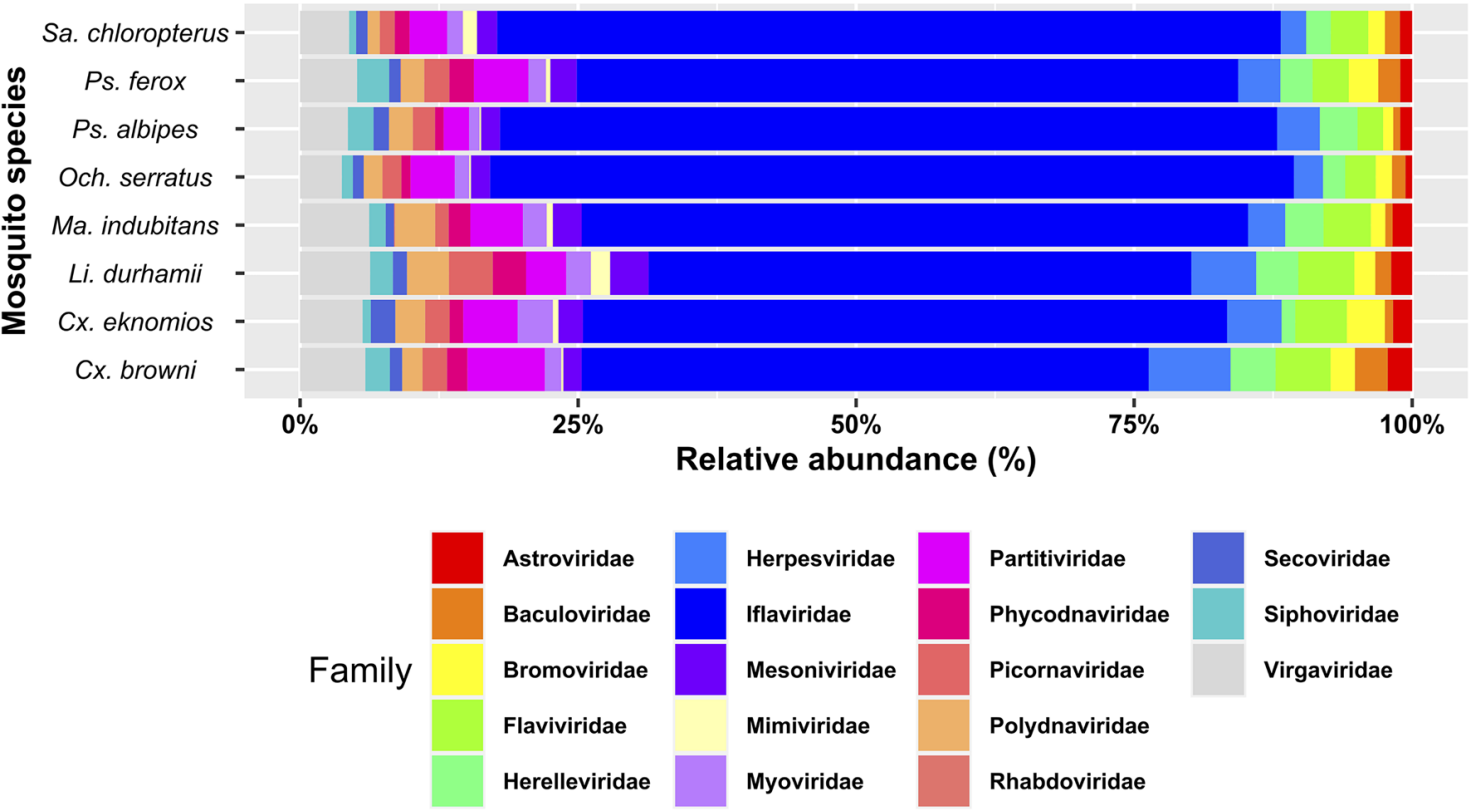
Relative abundance of viral families in each mosquito species. Bar graph showing virus families in the virome of each mosquito species *Och. serratus, Cu. eknomios, Cu. browni, Li. durhamii, Ma. indubitans, Ps. albipes, Ps. ferox, Sa. chloropterus.* The abundance of viral families was estimated by transforming the number of reads into relative values, providing an assessment of their presence in each mosquito species. Figure was created in R studio.

To obtain a comprehensive understanding of the virome present in the mosquitoes under study, we initially analyzed the identified viral families. For direct taxonomic assignment of the viral reads, we used the metagenomic sequence classifier Centrifuge, together with a high-quality virus database obtained from GenBank via NCBI. The metagenomic classification results were analyzed and visualized using the Pavian package. The virome in mosquitoes captured in the Orinoco region were mainly represented by RNA virus families known to contain ISVs and/or arboviruses. These included the families Iflaviridae, Baculoviridae, Mesoniviridae, Partitiviridae and Flaviridae (Fig. 1). Iflaviridae was the most abundant family in all mosquito vector pools analyzed. In addition, viral families infecting host plants were identified, such as Virgaviridae, Bromoviridae, Polydnaviridae and Secoviridae, as well as viral families infecting host bacteria, such as Herelleviridae, Siphoviridae and Myoviridae. Families associated with vertebrate viruses were also identified, the most common taxonomic group being Herpesviridae. Another representative family in the mosquito vector virome was Mimiviridae, mainly associated with infection of the protist host (Fig. 1).

### Mosquito virome includes viruses from a diverse range of hosts

**Figure 2:**
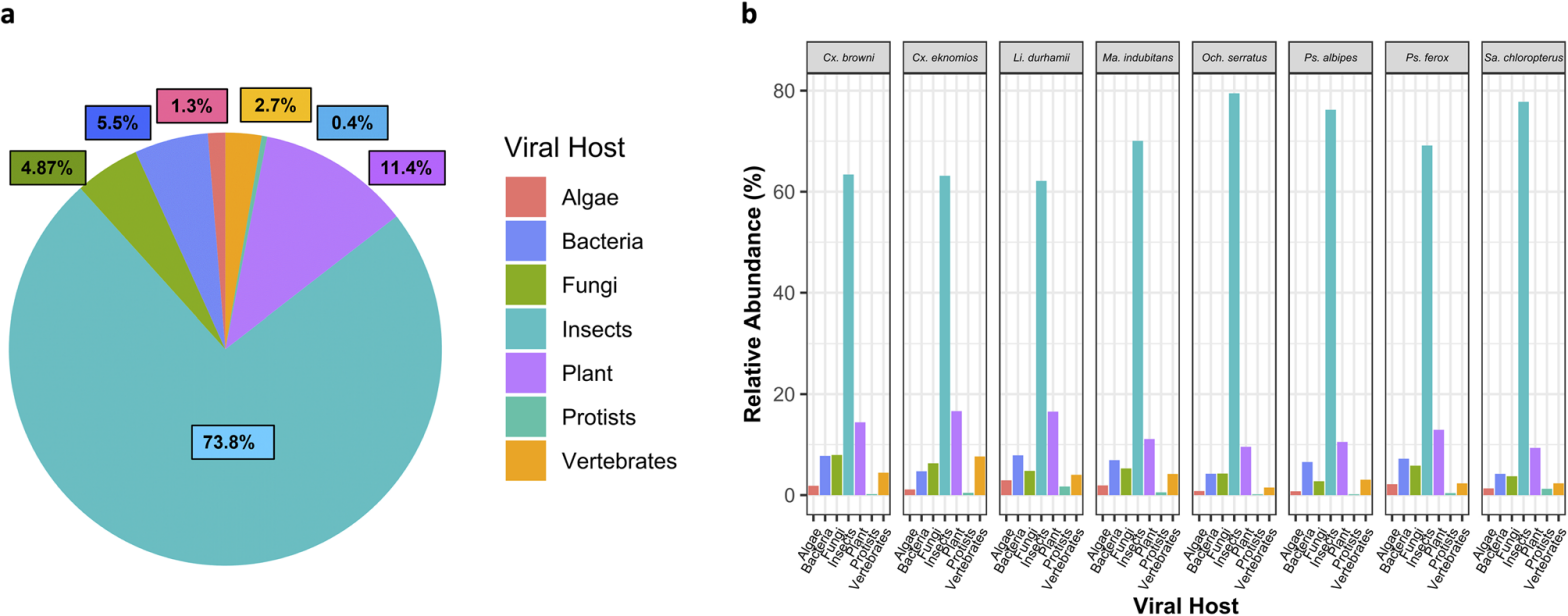
Hosts of viruses in the viromes of mosquitoes (Culicidae) in a rural ecosystem in Vichada, Colombia. (**a**) Proportions of viruses in different host groups from mosquitoes. (**b**) Relative abundance of viruses grouped by host organism in each vector species. To estimate the abundance, the number of reads was transformed into relative values, providing an assessment of their presence in each mosquito species. Figure was created in R studio.

Based on the data obtained from the taxonomic assignment of viral families, we performed a verification of the respective host species in the NCBI database. Our results revealed that mosquitoes of the species *Och. serratus, Cx. eknomios, Cx. browni, Li. durhamii, Ma. indubitans, Ps. albipes, Ps. ferox,* and *Sa. chloropterus* carried viruses belonging to various host groups. These included insect, vertebrate, plant, fungal (mycovirus), bacterial (bacteriophage), algal and protist viruses. Insect-specific viruses (ISVs) were the most abundant in all mosquito species, with 73.8% of the viruses identified. After ISVs, plant viruses accounted for 11.4% of the total, while bacterial viruses accounted for 5.5%, fungal viruses for 4.87%, vertebrate viruses for 2.7%, and viruses with algal and protozoan hosts for 1.34% **(**Fig. 2a). Among the mosquito species, *Och. serratus, Ps. albipes*, and *Sa. chloropterus* mosquitoes exhibited the highest percentages of insect-specific viruses (ISVs). On the other hand, mosquitoes belonging to the *Culex* genus (*Cx. eknomios* and *Cx. browni)* and *Li. durhamii* showed the highest proportion of plant viruses **(**Fig. 2b**).**

### Shared ISVs among different mosquito species

**Figure 3:**
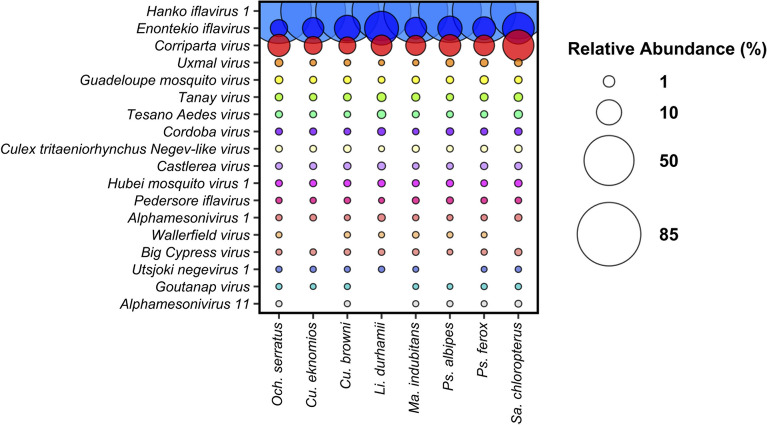
The relative abundance of Insect-Specific Virus (ISVs) in mosquito species. Bubble plot showing the relative abundance of ISVs*.* The abundance of viral species was estimated by transforming the number of reads into relative values, providing an assessment of their presence in each mosquito species. Figure was created in R studio.

To enhance the precision of taxonomic assignments for viruses in the studied mosquito vectors, we established a new NCBI-Mosquito virus database. This database was curated specifically for Culicidae (Diptera) hosts, incorporating all relevant sequences accessible on NCBI. The Centrifuge program and visual representation followed the same specifications described above for viral families. The analysis of viral species abundance involved the transformation of reads for each ISV into relative values for individual mosquitoes. This approach facilitated the assessment of viral presence in vector mosquitoes collected from the Orinoco River basin, revealing a common set of ISVs across all studied vector mosquitoes. Metaviromes were predominantly composed of *Hanko iflavirus 1*, which showed the highest abundance in all mosquito species. This ISV presented a high abundance in *Cx. eknomios* (88.4%) and *Och. serratus* (88.0%), whereas its abundance was lower in *Sa. chloropterus* (63.5%). In addition, *Enontekio iflavirus* ranked second in abundance in the mosquito virome, especially in *L. durhamii* (21%) and *Sa. chloropterus* (18%). Another important virus, *Corriparta virus*, showed the highest prevalence in *Sa. chloropterus* (16%) and the lowest in *Cx. browni* (3.5%) (Fig. 3).

In addition, other ISVs were identified in mosquito samples in smaller proportions, accounting for 1.3% of the total number of viruses identified. Although these ISVs were common to most of the groups evaluated, their abundance varied. *Uxmal virus, Guadalupe mosquito virus* and *Tanay virus* were the most prevalent in all mosquito samples. In contrast, *Tesano Aedes virus, Cordoba virus, Culex tritaeniorhynchus Negev-like virus, Castlerea virus, Hubei mosquito virus1, Pedersore iflavirus* and *Alphamesonivirus 1* showed lower abundance in the mosquito virome (Fig. 3). Statistical analysis, which employed the Kruskal–Wallis test followed by Dunn's post hoc test with Benjamini–Hochberg correction (*p value* > 0.05), showed no apparent differences in viral composition among the mosquito species studied. However, a notable distinction in the relative abundance of *Hanko iflavirus 1* was observed when compared to other viral species by this test (*p* value = 0.00019) (Figure S1).

## Discussion

Exploring the mosquito virome and unraveling how its composition influences arbovirus transmission is paramount to understanding the emergence of arboviral diseases and the dynamics of outbreaks^20,22,25^. In the present study, we performed metagenomic sequencing to characterize the virome of some epidemiologically important mosquito species associated with arbovirus transmission in rural and sylvatic cycles. The study was carried out in a local savanna ecosystem of the Colombian Orinoco region, an area with limited anthropogenic activity, which could be susceptible to spillover events under scenarios of anthropogenic intervention^41^. Specifically, we have focused on eight vector species, including *Och. serratus, Cx. eknomios, Cx. browni, Li. durhamii, Ma. indubitans, Ps. albipes, Ps. ferox,* and *Sa. chloropterus,* with potential to transmit viral agents causing diseases of public health importance^5,8,19^*.* These species were selected based on a previous study that identified them as representative mosquito species in terms of abundance and importance in the study area^41^.

Our investigations have revealed a predominance of insect-specific viruses (ISVs) in the virome composition of all mosquito species studied. The ISVs, well-documented as nonpathogenic viruses exclusive to insect hosts^20^, demonstrate no transmission to vertebrates, including humans^23,25^. Recent studies have underscored the significant potential of ISVs as biological control agents against vector-borne diseases. This influence is demonstrated through various mechanisms, encompassing both the enhancement and suppression of arbovirus replication within the mosquito vector. These mechanisms include acceleration of the extrinsic incubation period^42^, competitive exclusion^43^, induction of antiviral immune responses^26^, interference with transmission pathways^44^, cellular resistance, and modulation of insect behavior^45^. The high prevalence of common ISVs in the virome profile in all vector species studied highlights the importance of continuing to study this group of ISV (Figs. 2 and 3), especially their adaptations and potential applications as biological control agents against vector-borne disease as suggested by several authors^24,25,45^.

In addition, our research uncovered a diverse representation within the virome of the eight mosquito vector species, including viruses associated with plants, fungi, bacteria, vertebrates, algae, and protists (Fig. 2). These findings suggest that the virome of field-collected mosquitoes, especially in rural settings in tropical areas (with a low degree of anthropogenic intervention), could reflect intricate interactions with their natural environment^27,29,30^. Possibly this phenomenon is likely to be influenced by some factors, such as breeding and feeding sites as has been suggested by previous studies^30,46,47^. However, future research could systematically explore the influence of local environmental conditions, encompassing factors such as temperature, humidity, rainfall, vegetation, nutrition, and mosquito breeding water sources. This exploration would yield crucial information on viral habitat-related factors that may shape the transient or permanent composition of the mosquito virome^29,32^. Unfortunately, we have not collected comprehensive data on the environmental and ecological characteristics of the ecosystem studied, which lays the groundwork for future research on the relationship between specific habitat variables and the virome of mosquito populations.

In the present study, conducted in a rural ecosystem of Colombian Orinoco, viromes of mosquito species belonging to *Ochlerotatus, Culex, Limatus, Mansonia, Psorophora* and *Sabethes* genera showed the presence of common ISV species. The specific conditions of the shared environment could explain the similarities observed in the viromes of different mosquito species co-occurring in the same habitat. These conditions could potentially act as a selective pressure on mosquitoes, a concept proposed in a recent study in Colombia^40^. These findings are in line with metatranscriptomic analyses of the virome of *Culex* mosquitoes (*Cx. pipiens* and *Cx. torrentium*) in northern Europe^7^, which showed similar levels of viral diversity and numerous shared viruses between the two vector species. They are also consistent with the Metaviromic study in Greece, where viral similarity was observed between different species within the same genus and even between different mosquito genera^31^.Therefore, in our study, conducted in a savanna ecosystem, with specific ecological characteristics, including low anthropogenic activity, could offer similar conditions that can potentially favor the adaptation and persistence of certain viral species, among mosquitoes that coexist within the same endemic habitat.

These common ISVs between mosquitoes are objects of study have been frequently detected in *Culex* and *Aedes* mosquitoes^5^ and were recently described in a viral metagenomics study in *Ochlerotatus* species in Europe^48^. Additionally, another dominant virus was *Corriparta virus*, classified within the *Orbivirus* genus^49,50^ which includes arboviruses with a wide range of hosts^50^. Interestingly, ISVs found in lower abundance, such as *Guadeloupe mosquito virus* (GMV) and *Hubei mosquito virus1* (HMV1), have been described as part of the "core virome" in *Aedes* species^27–29^, indicating a broad global distribution. Meanwhile, other ISVs with low representation (Fig. 3), such as *Uxmal virus, Tanay virus, Cordoba virus,* and *Castlerea virus*, from the *Negevirus* genus, are phylogenetically related to plant viruses^51,52^, suggesting the adaptation of plant viruses in insects that feed on plant fluids, possibly through selection pressure processes^40^. It is noteworthy that the ISVs detected in this study have been reported with a wide geographic distribution and a broad range of mosquito host species, including *Aedes, Culex, Ochlerotatus,* and *Mansonia*^27,45,52,53^. It is crucial to continue studies that help elucidate the complex interactions involving virome shape in mosquitoes, especially the persistence of these common ISVs in vector species over time and geographic space. New insights in this area of study could reveal the critical role that some ISVs play in host fitness, especially in the presence and absence of viral pathogens.

In conclusion, our study provides a comprehensive insight into the viral composition within mosquitoes, collected from a local savanna ecosystem in the Colombian Orinoco. Despite the relatively stable nature of this ecosystem, the potential for spillover events increases significantly under scenarios involving anthropogenic intervention. Leveraging a metagenomic approach and advanced third-generation sequencing technologies, we identified the presence of insect-specific viruses (ISVs) common in the virome of these vector species. Furthermore, the virome of all mosquitoes also exhibited representation of viruses associated with plants, fungi, bacteria, vertebrates, algae, and protists. This broad viral representation underscores the intricate interactions of mosquitoes with their natural habitat. We propose further exploration of environmental and geographical conditions to enhance our understanding of the ecological dynamics of mosquito-vectors.

Finally, it is necessary to recognize some limitations of our study. First, the small number of samples could have limited the information on viral composition in the vector species studied. In addition, our investigation was based on a single sampling event of entomological material, and we did not collect detailed information on the environmental and ecological characteristics of the ecosystem studied, which could have provided valuable information on host-local environment interactions and their influence on virome.

We consider our findings to be an important contribution to the knowledge of the virome in Neotropical mosquito vectors described as potentially transmitting pathogenic viruses of the genera *Ochlerotatus, Culex, Limatus, Mansonia, Psorophora* and *Sabethes*. The presence of common ISVs in the virome of different vector species suggests that ecological information from the study site emerges as an essential component for understanding the determinants of virome in vectors. It highlights the need for further investigation of shared viruses among mosquito species, which would provide a more complete representation of diversity, distribution, and potential applications as control agents.

## Methods

### Study area and mosquito collection

**Figure 4:**
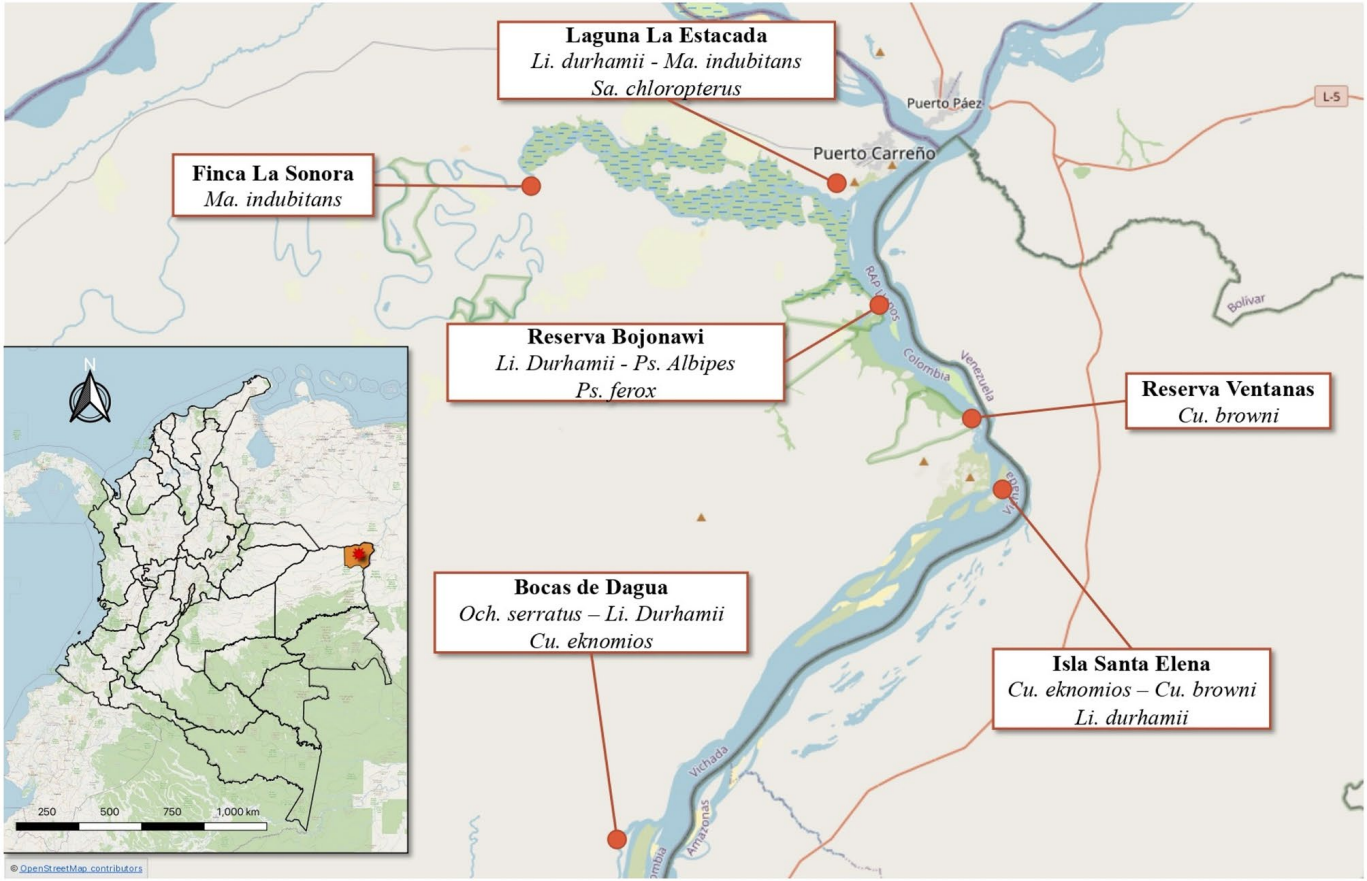
The map shows the geographic location of the Culicidae mosquito collection sites in the municipality of Puerto Carreño, located in the department of Vichada in Colombia. The georeferenced sampling points in different areas along the Orinoco River are highlighted, and each point is labeled with the local name of the corresponding sampling site and the mosquito species selected for viral metagenomic analysis. This map was produced using the QGIS 3.22.14 tool and different data sources were used, including Basemap: Esri Satellite World_Imagery (MapServer) available at https://bit.ly/3MRtYCf, as well as sources from Esri, Maxar, Earthstar Geographics, and the GIS user community. This map is licensed under CC BY-SA 3.0.

Based on the identification of mosquito species circulating in the rural area of the Eastern Plains of Colombia from a previous study conducted by our research group^41^, we selected eight adult mosquitoes of the species *Ochlerotatus (Aedes) serratus, Culex eknomios, Culex browni, Limatus durhamii, Mansonia indubitans, Psorophora albipes, Psorophora ferox, Sabethes chloropterus, Culex eknomios, Culex browni, Limatus durhamii, Mansonia indubitans, Psorophora albipes, Psorophora ferox,* and *Sabethes chloropterus*, for metagenomic sequencing belonging. Species selection was based on their abundance in the study area and per sampling site^41^ **(**Fig. 4), as well as their epidemiological importance for the transmission of arboviruses of public health importance^3,17,18^. Briefly, the collection of adult mosquitoes in the field was carried out at the beginning of the dry season (low water) in December 2020, in the rural area of the municipality of Puerto Carreño, in the department of Vichada. The study site was located in the Eastern Plains of the Orinoco region, bordering Venezuela. This ecosystem is characterized by an extensive grassland plain (savannah), composed mainly of grasses, with the presence of forest patches. Geographically, it is located at 6° 11′ 16″ north latitude and 67° 28′ 57″ west longitude, with an altitude of 51 m.a.s.l. (meters above sea level). The weather is tropical, with an average maximum temperature of 39.5 °C, rainfall of 2688 mm and an annual relative humidity of 70%. Sampling localities were chosen in proximity to the Orinoco River (Fig. 4), with shared ecological characteristics, particularly in a region influenced by recreational fishing and ecotourism, which create favorable conditions for mosquito breeding^41^. These areas also have nearby human settlements and have experienced minimal human interference, with limited disturbance or disruption due to human activity. This is of particular concern in view of possible future anthropogenic activities, which could facilitate the spread of potentially pathogenic viruses.

For mosquito collection, entomology professionals from the Department of Vichada Health Secretary provided support. A single sampling per site was carried out, lasting one hour per site, using mechanical vacuums during the day. In the forest patches, all mosquitoes were collected in the same field season. To avoid contamination of the biological material, gowns and gloves were worn throughout the handling process. In addition, trapping, storage, and transport material was pre-sterilized. Only adult mosquitoes were collected (males and females) and subsequently morphologically identified to species level using taxonomic keys. Mosquitoes of major entomovirological importance, belonging to the subfamily Culicinae, were selected at each sampling point, and grouped into pools of 2–6 individuals according to the species identified. In some cases, mosquitoes were stored individually for subsequent confirmation based on mitochondrial cytochrome oxidase I (COI) sequencing. Entomological material was subsequently preserved in RNA (DNA/RNA shield, Zymo. R1100-50) at − 4 °C. Finally, the samples were analyzed at the microbiology laboratory of the Universidad del Rosario in Bogotá, Colombia. The Hamilton Microlab Star automated system and the MagBead Quick-DNA/Viral RNA kit (Ref. R2141, Zymo Research) were used to extract RNA from pooled and individual entomological material, after homogenization of the entomological material using the TissueLyser II® tissue homogenizer (Qiagen, Hilden, Germany). Subsequently, RNA was quantified using the NanoDrop™ spectrophotometer (Thermo Fisher Scientific, Waltham, MA, USA) stored at − 80 °C.

### Sample preparation and viral enrichment

Initially, samples of the selected mosquito species were checked for RNA quality and concentration. Then, 15 pools were chosen to reflect the abundance of mosquitoes (Diptera) in the study area. Thus, the species *Och. serratus* species, due to its high prevalence, was represented with the highest number of pools, followed by *Ps. ferox* (Table 1). The RNA used for sequencing was obtained from the 2020 RNA stock. In this regard, most of the pools analyzed corresponded to RNA from mosquitoes that were previously grouped by species and subsequently subjected to RNA extraction. For each pool, we ensured that it was composed of RNA from five individuals of the same species. Subsequently, the Ribo-Zero Plus rRNA Depletion kit (Illumina, REF: 20036696) was used in the RNA pools, following the manufacturer's instructions. Next, viral enrichment was carried out using the Rapid-SMART9n methodology (Switching Mechanism at the 5′ end of RNA Template), according to Claro et al.^54^ with a minor modification. In summary, cDNA synthesis involved 5 μl of RNA, 0.5 μl of RLB-RT9N primer (TTTTTCGTGCGCCGCTTCAACNNNNNNNNN, 2 μM), and 0.5 μl of dNTPs (10 mM) (New England BioLabs, USA), followed by incubation at 65 °C for 5 min. A mix containing 2 μl of SuperScript IV First-strand Buffer, 0.5 μL of 0.1 M DTT, 0.5 μl RNase OUT, 0.5 μl RLB TSO (GCTAATCATTGCTTTTTCGTGCGCCGCTTCAACATrGrGrG, 2 μM), and 0.5 μL SuperScript IV (Invitrogen, Carlsbad, CA, USA) was then added to the annealed RNA. The mixture underwent an incubation at 42 °C for 90 min followed by 10 min at 70 °C to yield the cDNA. Next, the cDNA product was amplified using 6.25 μl of LongAmp Taq 2X master mix (New England BioLabs, USA), 4.875 μl of NFW, 0.125 μl of RLB primer (TTTTTCGTGCGCCGCTTCA, 20 μM), and 1.25 μl of cDNA, employing the following conditions: 98 °C for 45 s, 30 cycles of 98 °C for 15 s, 62 °C for 15 s, and 65 °C for 5 min, with a final step at 65 °C for 10 min. During both the cDNA construction and post-viral enrichment steps, all samples were quantified to ensure the proper execution of the process. This quantification was performed using the Qubit dsDNA High Sensitivity Assay (Life Technologies, USA) on the Qubit 3.0 instrument (Life Technologies, USA), following the manufacturer's instructions.

### Nanopore library preparation and viral sequencing

Third-generation technology was used for sequencing. Initially, the End Prep procedure was employed using the commercial NEBNext® Ultra™ II End Repair/dA-Tailing Module kit for end preparation. For barcode ligation, the NEBNext® Ultra™ II Ligation Module kit was used. Finally, the adapters were ligated with a NEBNext® Quick Ligation Module kit together with the Oxford Nanopore Technologies ligation kit (SQK-LSK109). To ensure removal of unligated barcodes and adapters, a cleaning step was performed with Beckman Coulter AMPure XP paramagnetic beads. The original library was sequenced on a MinION device from Oxford Nanopore Technologies using R.9.4 flow cells. The sequencing process followed the standard 48–72 h script with MinKNOW 1.15.1 software, allowing for accurate and efficient data generation.

### Bioinformatic analysis

For bioinformatics analysis, raw Fast5 files were subjected to base calling and demultiplexing using the Guppy V3.1.5 tool from Oxford Nanopore Technologies^55^. Low quality reads with a score below 7 were filtered out. The obtained set of long reads was then subjected to statistical analysis using the NanoStat V 1.1.2 tool (https://github.com/wdecoster/nanostat) to determine the average length and quality scores. Host filtering was not performed, as no reference genome is available for most of the mosquito vector species studied. To improve data accuracy, files were filtered to remove prokaryotic sequence contamination (bacterial and archaeal ribosomal reads) using Minimap 2.24 software^56^ (https://github.com/lh3/minimap2). This software was specifically designed to align long genomic reads obtained from Oxford Nanopore sequencing. Alignment was performed against the prokaryotic database SILVA_138.1 (https://www.arb-silva.de/documentation/release-1381/). Finally, the aligned reads were then converted into a sorted BAM file using SAMtools^57^(https://github.com/samtools/samtools) and Bam2fastq tools (https://github.com/jts/bam2fastq).

The clean sequences obtained after filtering were taxonomically assigned using the metagenomic sequence classifier Centrifuge tool v1.0.4^58^. The Centrifuge tool is used to count the number of reads that align to each taxon and produces a table with this information. To assign reads to specific viruses a custom Centrifuge indexing viral database was constructed from viral gene and genome sequences available from GenBank via NCBI Virus repositories (74) (https://www.ncbi.nlm.nih.gov/labs/virus/vssi/#/), until the most recent accession date in October 2022. The database contained 12,709 viral sequences that were selected based on completeness, non-redundancy (nr/nt), and no ambiguous characters. This viral database was initially used to get an overall representation of virus families in vector mosquitoes and verify the viral hosts. To deepen the taxonomic assignment results for the viruses present in the mosquito virome (ISV/arbovirus), a new NCBI-Mosquito virus database was constructed by refining the results based on the Culicidae (Diptera) host option, resulting in a total of 2899 viral sequences. In both cases, the database was selected based on completeness, non-redundancy (nr/nt) and absence of ambiguous characters. The taxonomic assignment in Centrifuge was performed utilizing a minimum length for partial hits (–min-hitlen) of 95 and a k classification parameter of 1. The resulting outputs were converted to Kraken-Report format using the Centrifuge-kreport function. The results of the metagenomic classification were further analyzed and visualized using the Pavian package^59^ (https://fbreitwieser.shinyapps.io/pavian/). To verify the assigned sequences, they were aligned against the non-redundant (nr) nucleotide database using the Basic Local Alignment Search Tool (BLASTn)^60^ considering parameters of percentage identity > 80%, E-value greater than 5 and minimum percentage of the coverage length of 60%. The number of reads assigned to viral families and species were converted into relative values to estimate their abundance within each mosquito species. Abundance barplots were generated using the ggplot2 package in RStudio^61^.

### Statistical analysis

To assess the normality of the data, the Shapiro–Wilk test was used, which indicated a non-normal distribution. To compare the abundance of viral taxa among the different mosquito species in the study, a non-parametric Kruskal–Wallis test for multiple comparisons was employed. For post-hoc analysis, Dunn's test with Benjamini–Hochberg stepwise correction was applied. This test was also used to compare viral species. Statistical analyses and data visualization were conducted using R software^61^. A significance level of *p*-value < 0.05 was considered statistically differential for all tests.

### Supplementary Information

Supplementary Figure 1.

## Data Availability

The raw sequence reads generated in this study are available at the NCBI Sequence Read Archive (SRA) database under BioProject PRJNA98633 (https://www.ncbi.nlm.nih.gov/bioproject/986331). BioSamples SAMN35839862-SAMN35839876.
