# Supplementary material for: Characterizing viral species in mosquitoes (Culicidae) in the Colombian Orinoco: insights from a preliminary metagenomic study
Source: Sci Rep. 2023 Dec 12;13:22081. doi: 10.1038/s41598-023-49232-9 (PMC10716246; doi:10.1038/s41598-023-49232-9)
Supplement: Supplementary file 1 — Supplementary Figure 1. [file 41598_2023_49232_MOESM1_ESM.pdf]

Characterizing Viral Species in Mosquitoes (Culicidae) in the Colombian Orinoco: Insights from a Preliminary Metagenomic Study

Marcela Gómez[1,2], David Martínez[1], Luisa Páez-Triana[1], Nicolás Luna[1], Jorge Luis De las salas[3], Carolina Hernández[1], Alexander Zamora Flórez[3], Marina Muñoz[1], Juan David Ramírez[1,4] *

[1] Centro de Investigaciones en Microbiología y Biotecnología-UR (CIMBIUR), Facultad de Ciencias Naturales, Universidad del Rosario, Bogotá, Colombia.

[2] Grupo de Investigación en Ciencias Básicas (NÚCLEO) Facultad de Ciencias e Ingeniería, Universidad de Boyacá, Tunja, Colombia.

[3] Secretaría Departamental de Salud del Vichada, Colombia.

[4] Molecular Microbiology Laboratory, Department of Pathology, Molecular and Cell-based Medicine, Icahn School of Medicine at Mount Sinai, New York, NY, USA

*Corresponding author:

E-mail: juand.ramirez@urosario.edu.co; juan.ramirezgonzalez@mssm.edu

Supplementary Information

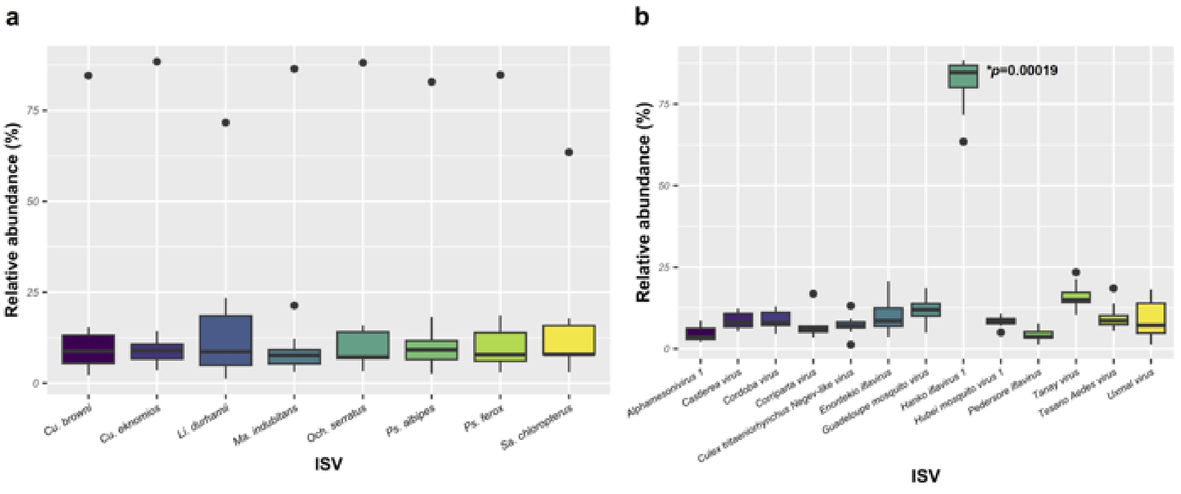

Supplementary Figure S1. Differential relative abundance of insect-specific viruses (ISVs) in the mosquito virome of a local ecosystem. Significant differences between **a.** mosquito species and **b.** ISVs identified in the mosquito virome were evaluated. Statistical differences are indicated with an asterisk (*) using the Kruskal-Wallis test; post-hoc analysis was conducted using Dunn's test with Benjamini-Hochberg correction at a 95% confidence level and a significance level of $p < 0.05$. The figure was created using R Studio.
